# ^1^H NMR Approach for Evaluating the Effects of a Natural Detergent on Olive Trees Infected by *Xylella fastidiosa* subsp. *pauca*

**DOI:** 10.3390/plants15071109

**Published:** 2026-04-03

**Authors:** Miriana Carla Fazzi, Chiara Roberta Girelli, Francesco Paolo Fanizzi

**Affiliations:** Department of Biological and Environmental Sciences and Technology, University of Salento, 73100 Lecce, Italy; mirianacarla.fazzi@unisalento.it (M.C.F.); fp.fanizzi@unisalento.it (F.P.F.)

**Keywords:** Olive Quick Decline Syndrome, NMR spectroscopy, metabolomics, multivariate statistical analysis, sustainability, Cellina di Nardò (*Olea europaea* L., Oleaceae)

## Abstract

*X. fastidiosa* subsp. *pauca* (*Xfp*) is the etiological agent of “Olive Quick Decline Syndrome” (OQDS). Cellina di Nardò (*Olea europaea* L., Oleaceae), one of the major Salento cultivars, is highly susceptible to *Xfp*, usually showing acute symptoms after infection. NuovOlivo^®^ a plant-derived formulation made with vegetal oils and water infusion from multi botanical species has been reported as effective against OQDS in plants affected by *Xfp*. A non-targeted ^1^H NMR (Nuclear Magnetic Resonance) fingerprinting approach, with unsupervised and supervised analysis, was applied to observe the possible changes in the metabolic profile in leaf samples of cultivars Cellina di Nardò naturally affected by *Xfp* treated with NuovOlivo^®^ compared to untreated plants. The major differences were observed for the content of quinic acid, malate, mannitol, glucose, oleuropein, and aldehyde derivatives in treated compared to untreated samples. The resulting data indicated a season-dependent plant response to both disease and treatment. Moreover, the overall differences observed between the two investigated years, suggest a general decrease in the differences for the discriminating metabolites over time. The protocol NuovOlivo^®^ was demonstrated to promote changes in the metabolic profile of olive leaves, suggesting a possible role of this treatment, integrated with good agricultural practices, against *Xfp* and OQDS.

## 1. Introduction

In 2013, *Xylella fastidiosa*, classified as subspecies *pauca* (*Xfp*), a xylem-limited Gram-negative Gammaproteobacterium, appeared in olive (*Olea europaea* L., Oleaceae) tree orchards of Salento (South of Apulia, Italy), causing a serious agricultural and heritage loss, strongly affecting the local farmers economically [1,2,3].

*X. fastidiosa* subsp. *pauca* is the etiological agent of “rapid olive decline complex” (“complesso del disseccamento rapido dell’olivo”, Co.Di.R.O.) previously known as “Olive Quick Decline Syndrome” (OQDS) [4]. The olive tree disease symptoms include yellow and brown lesions on leaf tips and margins, extensive twig dieback, and consequent tree mortality [5]. The plants affected by *Xfp* are characterized by a reduced olive production and lower fruit quality because of xylem blocking and decreased water and nutrient flux, often resulting in further death of the plants [6,7,8].

The major Salento cultivars that are particularly sensible to *Xfp*, usually showing acute symptoms after infection, are Ogliarola Salentina and Cellina di Nardò [9]. These cultivars are also well-known as basic constituents of the local PDO (Protected Designation of Origin) Extra Virgin Olive Oil (EVOO) “Terra d’Otranto” and for both their economical and landscape relevance, representing part of the Salento history [6].

The occlusion of xylematic vessels due to *Xfp* biofilm inside the infected plants is considered the basic cause of the disease symptoms. According to specific indications for a quarantine pathogen infection, the eradication of both infected and possibly infected plants, aimed at bacteria elimination, represented the only stated procedure for disease treatment [3,10].

Presently, control strategies include the application of integrated and preventive approaches, such as the use of varieties that are tolerant or resistant to the *Xfp,* and targeted agronomic practices [11]. Specific anatomic features of different cultivars have been related to the observed tolerance to infection [12]. For example, Cellina di Nardò is characterized by larger and less numerous xylematic vessels than Leccino, resulting in a higher vulnerability to the disease [13]. Moreover, both Ogliarola Salentina and Cellina di Nardò exhibited a reduced concentration of phenolic compound (such as hydroxytyrosol derivatives), which can play a role in the defence response against *Xfp*, when compared to Leccino [14].

Thus, after the Co.Di.R.O. outbreak in Salento, specific investigations into the resistance and resilience of other local (Salento and Apulia) cultivars were promoted, with the aim of their potential use in new groves to counteract the disease [15]. Consequently, in 2017, after several studies conducted on the total infected area of Salento, the cultivars Leccino and FS-17 (Favolosa^®^, a selection of seedlings of the Frantoio cultivar) were defined as “resistant” and were indicated for possible new cultivations [16]. Among the suggested reasons for the exhibited resistance to *Xfp*, the activation of various metabolic responses in infected plants have been extensively investigated. For example, in the olive cultivar Leccino, lignin accumulation in the xylem tissue appeared to be relevant for pathogen tolerance [17], while the cultivar FS-17 presents a genotype with more resistance traits [18,19]. Furthermore, recent declarations from specific regional administrative offices [20] stated that, besides Leccino and FS-17, Lecciana and Leccio del Corno can also be considered as less sensible to *Xfp* than susceptible or highly susceptible cultivars. Therefore, to date, only four cultivars (Leccino, FS-17, Lecciana, and Leccio del Corno) are specifically indicated for new cultivations in the infected areas.

In recent years, besides research aimed at finding *Xfp*-tolerant/resistant cultivars, some chemical treatments have also been investigated and found possibly useful in terms of pathogen control in autochthonous susceptible varieties, including Ogliarola Salentina and Cellina di Nardò [3,10]. In recent years, several studies have also focused on the investigation of possible treatments against *Xfp*. Recently, Del Grosso et al. performed a high-performance in vitro investigation of the antimicrobial activity of various compounds used against *Xfp* and also found some interesting results regarding a specific citric acid Zn and Cu micronutrient formulation (Dentamet^®^) [21]. In another recent study, the antimicrobial and bactericidal activities of some commercial fertilizers, which contain metal ions such as copper and/or zinc, complexed with phosphites and bioavailable silicon, was demonstrated in vitro [22]. Furthermore, it has been demonstrated that phenolic compounds, alone or in combination with treatments, could inhibit the increase in different bacterial species [23,24]. In particular, phenolic compounds in olive leaf extracts, such as oleuropein, have been described as particularly potent and promising natural agents against *Xfp* infection [11,25]. These results also suggest that specific treatments, together with agronomic and phytosanitary control measures, can be applied to contrast the pathogen, significantly reducing the severity of the disease and improving yield in a sustainable way [22].

NuovOlivo^®^ a plant-derived formulation made with plant oils and water infusion from multi botanical species has also been reported as effective against OQDS in plants affected by *Xfp* [6,26,27]. The activity of this latter commercially available preparation is likely to be related to its diverse phytochemical components, including primary and secondary plant metabolites and phytohormones [6]. Both Dentamet^®^ and NuovOlivo^®^ have been recently listed by Portaccio et al. in a summary of research examining the efficacy of different treatments against *Xfp* utilizing organic compounds, synthetic molecules, and salt or metal-based formulations [11]. Extensive research, including metabolomics studies, reported the effects of Dentamet^®^ treatments when used in the field for OQDS control in plants affected by *Xfp* [3,10,11,21,22,28,29,30].

In the present work we investigated metabolic alterations in olive trees, induced using the NuovOlivo^®^ product in one of the most representative and highly susceptible varieties to *Xfp* in the Salento area, with the aim of obtaining useful indication of the treatment’s potential [9,29,30,31,32]. A non-targeted ^1^H NMR (Nuclear Magnetic Resonance) fingerprinting approach, with unsupervised and supervised analysis, was applied to observe the possible changes in metabolic profile in leaf samples of the Cellina di Nardò cultivar naturally affected by *Xfp* treated with NuovOlivo^®^ compared to untreated plants.

## 2. Results

### 2.1. Unsupervised PCA and Supervised Pairwise OPLS-DA Introductory Analyses: Total Dataset

**Figure 1:**
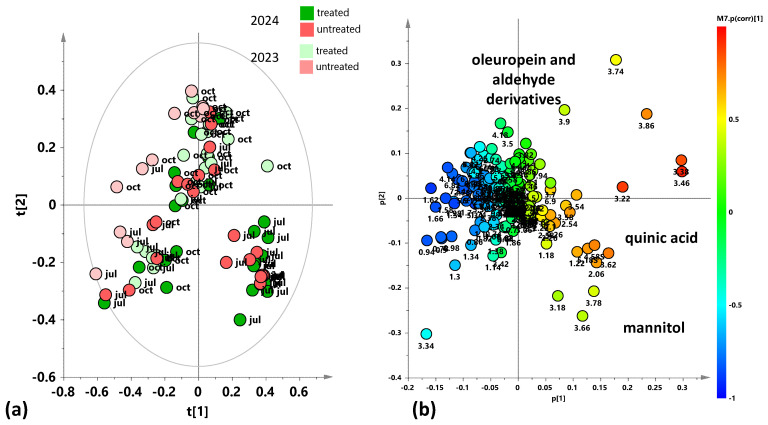
(**a**) PCA t[1]/t[2] score plot for the whole dataset of leaf samples (five components give R^2^X (cum) = 0.809, Q^2^(cum) = 0.628). (**b**) Loading scatter plot for the PCA model (**a**) coloured according to the correlation scaled coefficient (p(corr) ≥ |0.5|). The colour bar associated with the plot indicates the correlation of the segregated metabolites between groups.

With the purpose of detecting a possible natural grouping of the total dataset of leaf sample extracts, an unsupervised PCA analysis was applied to the ^1^H-NMR spectra-derived data. In the resulting PCA model (R^2^X(cum) = 0.809, Q^2^(cum) = 0.628), the total variance of 80.9% was explained through five components describing the samples’ distribution in the model space (t[1], t[2], t[3], t[4], and t[5], accounting for 32%, 22.1%, 12.5%, 7.37%, and 6.89% of the total variance, respectively). The t[1] and t[2] PCA score plots are reported in Figure 1a. The exploratory unsupervised analysis of the total dataset shows a partial differentiation according to the considered year along t[1], more marked for the samples occurring at the negative compared to the positive t[2] values. On the other hand, the discrimination along the t[2] component appears clearly related to the sampling season (October vs. July for the positive and negative t[2] values respectively). The identified bucket-related signals, representative of specific metabolites, accountable for the position of the samples in the PCA score plot, are reported in the loading plot of Figure 1b. In particular, different levels of glucose (3.18 ppm, 4.58 ppm, and 5.18 ppm), quinic acid (2.06 ppm), mannitol (3.66 ppm, 3.78 ppm, 3.86 ppm, and 3.9 ppm), as well as oleuropein and its aldehyde derivatives (7.54ppm, 5.9 ppm), are responsible for the differentiation preliminarily observed through the PCA analysis. The unsupervised analysis also suggests the need for further specific investigation focused on the differences between treated and untreated samples due to their year and season dependence.

**Figure 2:**
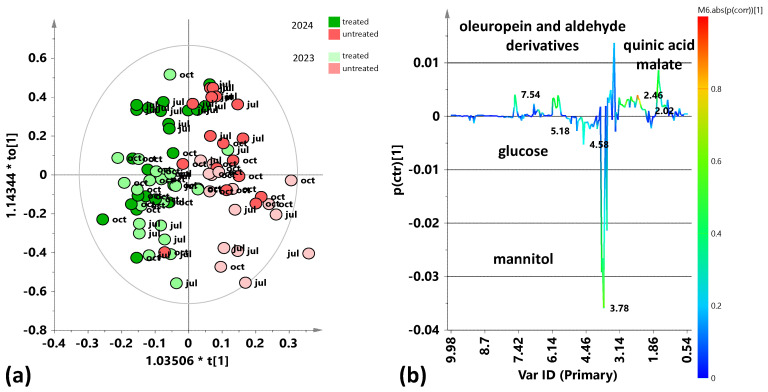
Pairwise OPLS-DA analysis comparisons between the treated and untreated leaf samples coming from all samplings (July 2023, October 2023, July 2024, and October 2024). (**a**) OPLS-DA score plot 1 + 2 + 0 components; R^2^X(cum) = 0.494; R^2^Y(cum) = 0.619; Q^2^ = 0.487. (**b**) S-line for the model coloured according to the correlation scaled coefficient (p(corr) ≥ |0.5|). The colour bar associated with the plot indicates the correlation of the segregated metabolites between groups.

For this reason, a supervised OPLS-DA analysis was performed to discriminate the treated and untreated samples within the entire dataset (July 2023, October 2023, July 2024, and October 2024 samplings). The score plot of the resulting model (Figure 2a), shows a reasonable separation of the treated samples compared to untreated samples across all years and both samplings’ seasons, along the predictive component with almost acceptable model parameters (R^2^X(cum) = 0.494; R^2^Y(cum) = 0.619; Q^2^(cum)= 0.487). By examining the corresponding S-line plot (Figure 2b), the discriminating metabolites responsible for differentiations were identified. Specifically, this model shows that all treated samples (coming from all samplings for both years) are characterized by high relative content of mannitol. On the contrary, the untreated samples (coming from all samplings across both years) have a higher relative content of oleuropein and aldehyde derivatives and malate. Moreover, interestingly, a clear further intra-class differentiation, essentially related to the sampling year, could be observed along the first orthogonal component specifically for the untreated samples.

### 2.2. Supervised Pairwise OPLS-DA Analysis: Treated 2023 vs. Untreated 2023 and Treated 2024 vs. Untreated 2024

Subsequently, we further investigated the differences between the treated and untreated leaf samples specifically for each of the two different years.

**Figure 3:**
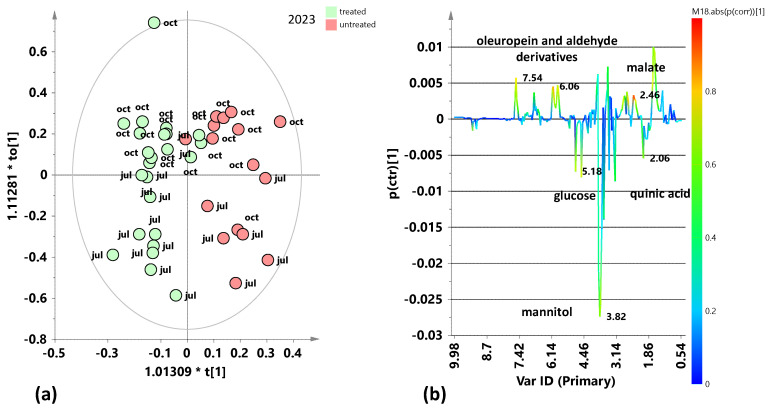
Pairwise OPLS-DA analysis comparisons between the treated and untreated leaf samples coming from the two samplings from 2023 (July 2023 and October 2023). (**a**) OPLS-DA score plot 1 + 2 + 0 components; R^2^X(cum) = 0.695; R^2^Y(cum) = 0.75; Q^2^ = 0.597. (**b**) S-line for the model coloured according to the correlation scaled coefficient (p(corr) ≥ |0.5|). The colour bar associated with the plot indicates the correlation of the segregated metabolites between groups.

The supervised OPLS-DA analysis relating to leaf samples coming from the two samplings of 2023 (July 2023 and October 2023) (Figure 3a) shows a good separation of treated compared to untreated samples along the predictive component with almost satisfactory model parameters (R^2^X(cum) = 0.695; R^2^Y(cum) = 0.75; Q^2^ = 0.597). The corresponding S-line plot (Figure 3b) indicates that the treated samples are characterized by high relative content of mannitol, glucose, and quinic acid, while the untreated samples show a higher relative content of oleuropein and aldehyde derivatives and malate. Moreover, it is possible to observe a clear intra-class separation according to the seasonality of the two samplings, with negative values (July 2023) and positive values (October 2023) along the first orthogonal component.

**Figure 4:**
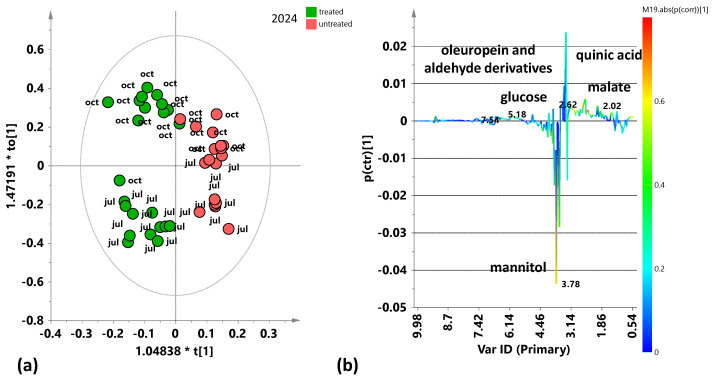
Pairwise OPLS-DA analysis comparisons between the treated and untreated leaf samples coming from the two samplings from 2024 (July 2024 and October 2024). (**a**) OPLS-DA score plot 1 + 2 + 0 components; R^2^X(cum) = 0.614; R^2^Y(cum) = 0.82; Q^2^ = 0.561. (**b**) S-line for the model coloured according to the correlation scaled coefficient (p(corr) ≥ |0.5|). The colour bar associated with the plot indicates the correlation of the segregated metabolites between groups.

Also, the subsequent supervised OPLS-DA analysis relating to the 2024 leaf samples (July 2024 and October 2024) (Figure 4a) displays a good separation of treated and untreated samples along the predictive component with satisfactory model parameters (R^2^X(cum) = 0.614; R^2^Y(cum) = 0.82; Q^2^ = 0.561). The corresponding S-line plot (Figure 4b) showed a higher relative content of mannitol in treated samples, while the untreated samples are characterized by a higher relative content of organic acids (malate). Furthermore, a good intra-class separation was also observed for the 2024 samples according to the sampling seasonality with negative (July 2024) and positive values (October 2024) along the first orthogonal component. Moreover, specifically for this year, the observed seasonal intra-class separation appeared clearly more marked for treated compared to untreated samples.

### 2.3. Unsupervised PCA and Supervised Pairwise OPLS-DA Analyses: Comparisons of Treated vs. Untreated in Single Sampling

**Figure 5:**
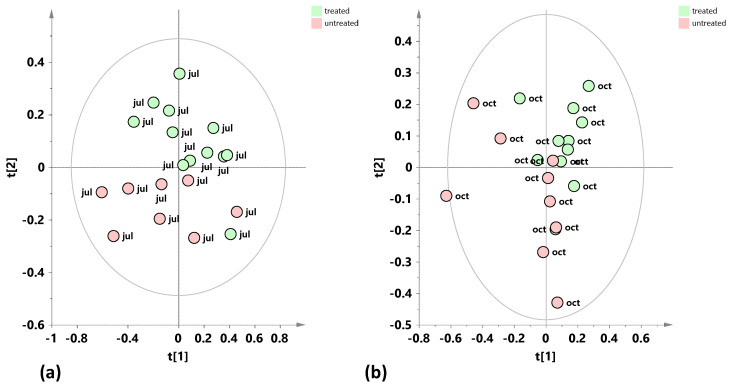
(**a**) PCA t[1]/t[2] score plot for the dataset of leaf samples relating to sampling of July 2023 (five components give R^2^X (cum) = 0.909, Q^2^ (cum) = 0.749). (**b**) PCA t[1]/t[2] score plot for the dataset of leaf samples relating to the sampling of October 2023 (five components give R^2^X (cum) = 0.872, Q^2^ (cum) = 0.578).

The differences between the treated and untreated leaf samples were further investigated at the single sampling level (July and October) for each of the studied years (2023–2024). The first sampling covered by this work was carried out in July 2023, followed by the subsequent sampling in October. An unsupervised PCA analysis performed on the NMR data obtained for each of the samples sets (July and October 2023) allowed for the exploration of their distribution related to the greater or lesser similarity of their metabolic profiles and highlighted the possible presence of clusters. The model in Figure 5a shows a clear natural separation along the second principal component t[2] (*y*-axis) between the treated and untreated July 2023 leaf samples. The further unsupervised model (Figure 5b), obtained for the October 2023 leaf samples, confirmed the separation between treated and untreated trees also for these samples, as already observed for the previous July sampling. In this case (October 2023 samples), differentiation was evident along both the first t[1] and the second t[2] principal components. Therefore, in both cases (July and October 2023), the PCA showed a natural separation between samples of leaf extracts from olive trees treated and untreated with the product.

**Figure 6:**
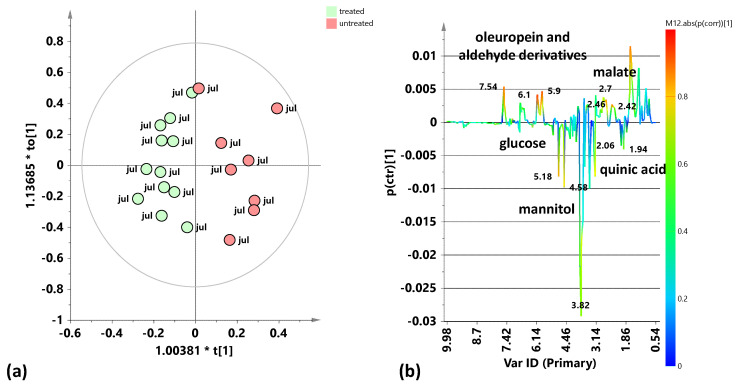
Pairwise OPLS-DA analysis comparisons between the treated and untreated leaf samples coming from the sampling of July 2023. (**a**) OPLS-DA score plot 1 + 2 + 0 components; R^2^X(cum) = 0.723; R^2^Y(cum) = 0.797; Q^2^ (cum) = 0.385. (**b**) S-line for the model coloured according to the correlation scaled coefficient (p(corr) ≥ |0.5|). The colour bar associated with the plot indicates the correlation of the segregated metabolites between groups.

To further investigate the specific differences between each group in relation to treatment, the supervised OPLS-DA analysis was then performed for both the July and October 2023 leaf samples, also taking into account the class membership (treated/untreated) of the samples into their respective models. The supervised OPLS-DA model for the July 2023 leaf samples (Figure 6a) shows, more clearly than the unsupervised analysis (PCA), a good separation along the predictive component ([t1]) between the samples from treated and untreated trees. The model parameters, obtained using the predictive and two orthogonal components (1 + 2), are characterized by satisfactory descriptive and positive, although low, predictive indicators: R^2^X(cum) = 0.723; R^2^Y(cum) = 0.797; and Q^2^ (cum) = 0.385. The molecular components responsible for class separation have been indicated in the S-line plot for the model in Figure 6b. Leaf samples from treated trees are characterized by a higher relative content of glucose (3.18, 4.58, and 5.18 ppm) and mannitol (3.82 ppm), compared to samples from untreated trees. However, the latter are characterized by a higher relative content of polyphenols, particularly oleuropein and its aldehyde derivatives (1.66, 5.9, 6.1, and 7.54 ppm). Furthermore, in the group of samples from treated plants, the presence of quinic acid (2.06 ppm)—a lignin precursor already indicated as a disease marker for olive trees infected by *X. fastidiosa* subsp. *pauca* [9]—was observed.

**Figure 7:**
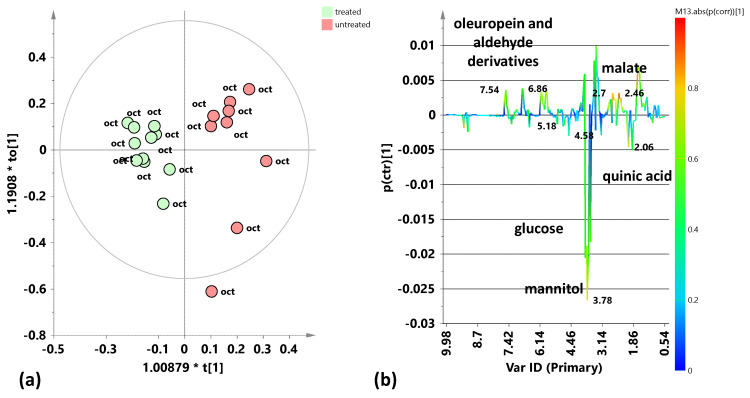
Pairwise OPLS-DA analysis comparisons between the treated and untreated leaf samples coming from the sampling of October 2023. (**a**) OPLS-DA score plot 1 + 2 + 0 components; R^2^X(cum) = 0.643; R^2^Y(cum) = 0.887; Q^2^(cum) = 0.691. (**b**) S-line for the model coloured according to the correlation scaled coefficient (p(corr) ≥ |0.5|). The colour bar associated with the plot indicates the correlation of the segregated metabolites between groups.

A supervised OPLS-DA model was also built for the October 2023 samples (Figure 7a). In this case too, good separation was observed along the predictive component between the two classes of samples related to treatment application or lack thereof. The model parameters, obtained using the predictive and two orthogonal (1 + 2) components, are satisfactory: R^2^X(cum) = 0.643; R^2^Y(cum) = 0.887; and Q^2^ (cum) = 0.691. The molecular components responsible for class separation result from the S-line plot of the model in Figure 7b. The leaf samples from the treated trees are characterized by a higher relative content of mannitol (3.78, 3.66 ppm) compared to the samples from the untreated trees. The latter, instead, are characterized by a higher relative content of polyphenols, particularly oleuropein, its aldehyde derivatives (7.54, 6.82, 5.86, 1.7 ppm), and malate (2.46 ppm). Additionally, in this case, a higher presence of quinic acid (2.06 ppm) was observed in the treated olive trees compared to the untreated ones. Therefore, the trend of metabolic profiles for the October 2023 samples generally shows almost the same differences between treated and untreated leaf samples previously observed for the July 2023 samples. Furthermore, a slightly higher intra-class variability (detectable along the first orthogonal component in the OPLS-DA scores plot) was observed, in particular, in the treated tree samples from the 2023 summer sampling (July) compared to the autumn one (October).

**Figure 8:**
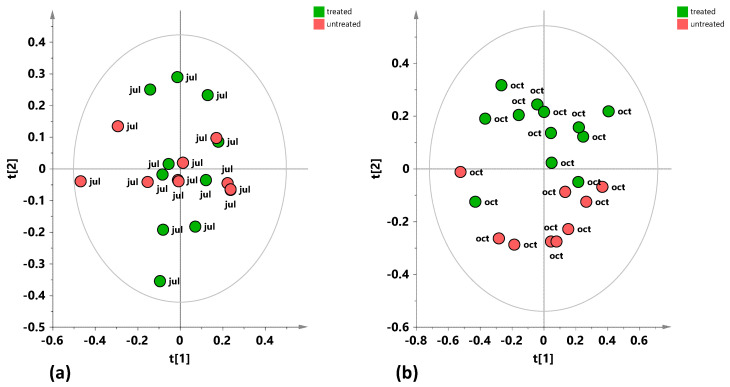
(**a**) PCA t[1]/t[2] score plot for the dataset of leaf samples relating to the sampling of July 2024 (five components give R^2^X (cum) = 0.832, Q^2^ (cum) = 0.492). (**b**) PCA t[1]/t[2] score plot for the dataset of leaf samples relating to the sampling of October 2024 (five components give R^2^X (cum) = 0.893, Q^2^ (cum) = 0.654).

The same analyses were then performed for the 2024 samples, both for the July and October samplings. The models for the unsupervised PCA analyses (Figure 8a, b) display a better separation between treated and untreated tree samples for the October (Figure 8b) samples compared to the July samples (Figure 8a), in particular along the second principal component, t[2].

**Figure 9:**
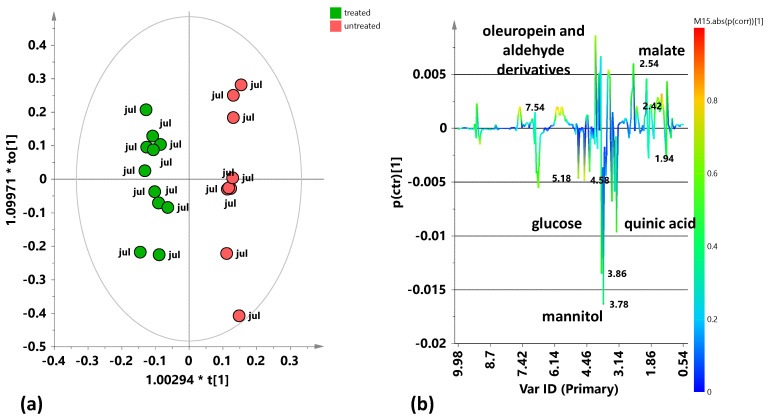
Pairwise OPLS-DA analysis comparisons between treated and untreated leaf samples coming from the sampling of July 2024. (**a**) OPLS-DA score plot 1 + 2 + 0 components; R^2^X(cum) = 0.503; R^2^Y(cum) = 0.973; Q^2^ (cum) = 0.911. (**b**) S-line for the model coloured according to the correlation scaled coefficient (p(corr) ≥ |0.5|). The colour bar associated with the plot indicates the correlation of the segregated metabolites between groups.

Nevertheless, the supervised OPLS-DA model for the July 2024 leaf extract samples (Figure 9a) indicate a good separation along the first component (predictive component [t1]) between the treated and untreated trees. The model parameters, obtained using the predictive and two orthogonal components (1 + 2), are quite satisfactory: R^2^X(cum) = 0.503; R^2^Y(cum) = 0.973; and Q^2^ (cum) = 0.911. The molecular components responsible for the class separation could be identified according to the S-line plot of the model (Figure 9b). The leaf samples from the treated trees are characterized by a higher relative content of α/β glucose (3.22, 3.46, 4.58 and 5.18 ppm) and mannitol (3.78, 3.82 ppm) compared to the untreated ones. On the contrary, the untreated leaf samples show higher relative content of polyphenols, particularly oleuropein and its aldehyde derivatives (6.02, 7.5, 9.22 ppm), as already observed in the July 2023 sampling.

**Figure 10:**
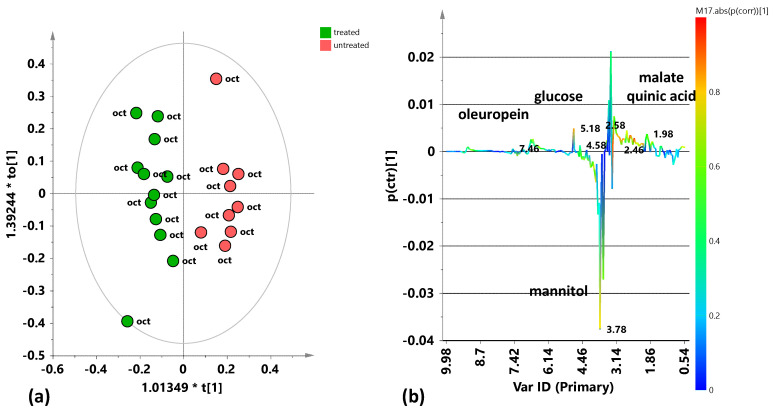
Pairwise OPLS-DA analysis comparisons between the treated and untreated leaf samples coming from the sampling of October 2024. (**a**) OPLS-DA score plot 1 + 2 + 0 components; R^2^X(cum) = 0.701; R^2^Y(cum) = 0.904; Q^2^ (cum) = 0.735. (**b**) S-line for the model coloured according to the correlation scaled coefficient (p(corr) ≥ |0.5|). The colour bar associated with the plot indicates the correlation of the segregated metabolites between groups.

According to the clear separation already observed in the PCA score plot (Figure 8b), the supervised OPLS-DA model for leaf samples of October 2024 (Figure 10a) shows a good differentiation along the predictive component between the treated and untreated trees. The model parameters, obtained using the predictive component and two orthogonal (1 + 2) components, resulted satisfactory also in this case: R^2^X(cum) = 0.701; R^2^Y(cum) = 0.904; and Q^2^ (cum) = 0.735. The metabolites responsible for class discrimination, indicated in the corresponding S-line plot (Figure 10b), increased organic acid malate and glucose for the untreated samples, while treated samples showed a higher relative content of mannitol. Similar differences were already noted in the supervised OPLS-DA analysis of the entire 2024 dataset, comparing treated and untreated samples of both treatments (July and October 2024) (Figure 4a, b).

### 2.4. Comparisons of Treated vs. Untreated: General Results

**Figure 11:**
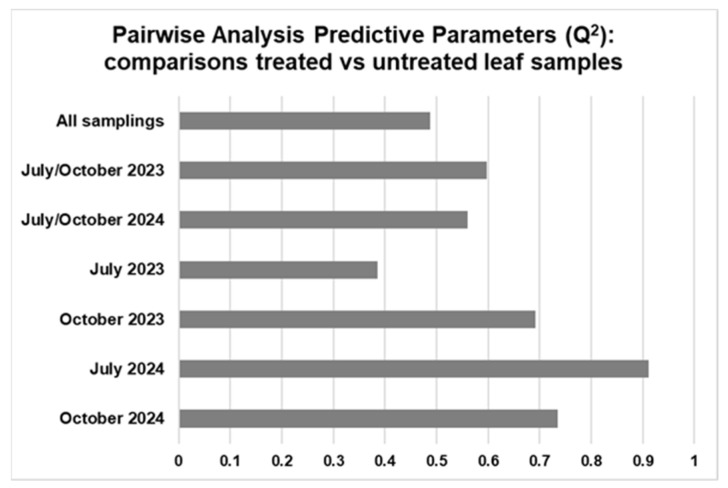
Bar graph of Q^2^ statistical parameter values of the OPLS-DA models for the comparison of treated vs. untreated leaf samples. Q^2^ is a predictive parameter of goodness t used to assess the robustness of the pairwise OPLS-DA analysis models. One predictive and two orthogonal components were considered for each model.

In all the pairwise comparisons between treated and untreated leaf samples, a generally good separation along the predictive component could be observed for all the investigated combinations, as proved by the resulting positive predictive (Q^2^) parameters values of the models (Figure 11). This value exceeds the significance value of 0.5 in most cases, ranging from a minimum of 0.385 to a maximum of 0.911 obtained for the selective treated vs. untreated comparisons of July 2023 and July 2024, respectively.

On the other hand, the Q^2^ results in the treated vs. untreated comparisons across the cumulatively considered samplings are quite similar between different years and when focusing on the complete dataset. Thus, to further finally assess the differences between the treated and untreated samples, the relative content of the discriminating metabolites was examined for each of the single samplings and, cumulatively, for each of the two investigated years, as well as for the entire dataset.

The identified discriminant metabolites of treated and untreated leaf samples were considered, and the fold change ratios (FC), calculated from the bucket integrals related to specific NMR signals, in the pairwise OPLS-DA models (Figure 2, Figure 3, Figure 4, Figure 6, Figure 7, Figure 9 and Figure 10). Statistically significant (*p* value < 0.05) differences between discriminating metabolites were obtained and considered for each pairwise comparison. Significant discriminating metabolites (|p(corr)| ≥ 0.5 and VIP ≥ 1 in at least one sampling) were chosen for the calculation of FC ratios and comparisons.

**Figure 12:**
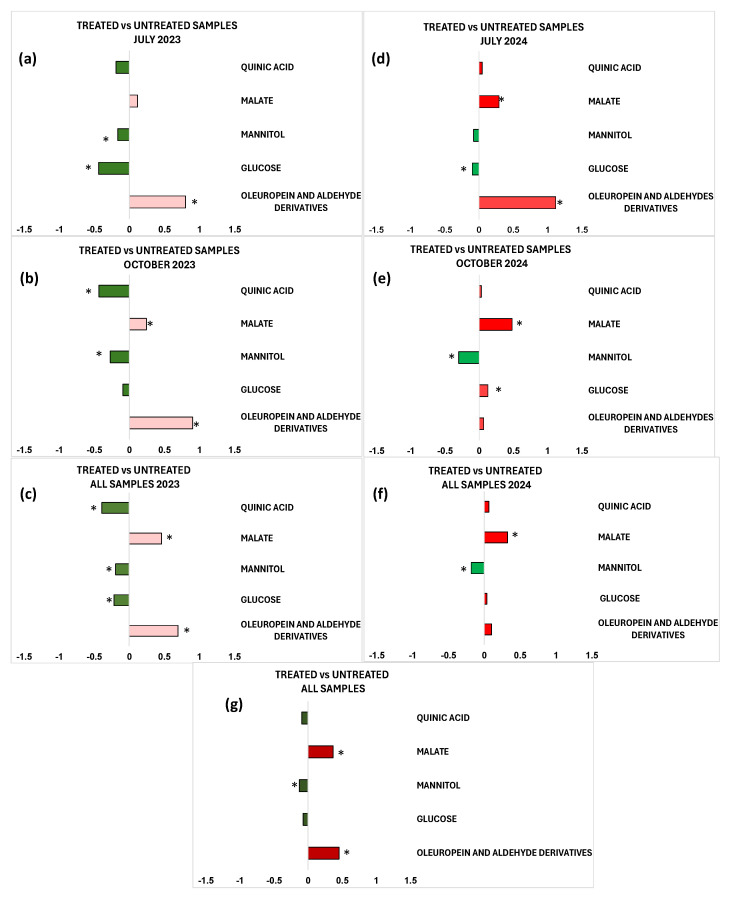
Graphic summary of the quantitative differences in discriminating metabolites for the comparison of treated vs. untreated extract samples in single sampling. (**a**) Treated vs. untreated leaf samples coming from the sampling of July 2023. (**b**) Treated vs. untreated leaf samples coming from the sampling of October 2023. (**c**) Treated vs. untreated for the whole dataset of leaf samples from 2023. (**d**) Treated vs. untreated leaf samples coming from the sampling of July 2024. (**e**) Treated vs. untreated leaf samples coming from the sampling of October 2024. (**f**) Treated vs. untreated for the whole dataset of leaf samples from 2024. (**g**) Treated vs. untreated for the whole dataset of leaf samples. Strong discriminating metabolites (|p(corr)| ≥ 0.5 and VIP ≥ 1 for at least one sampling) were chosen for fold change ratios comparisons. The *x*-axis reports log_2_ (fold change) values. The asterisk (*) indicates statistical significance at *p* < 0.05 of metabolites.

In particular, the important NMR signals for quinic acid, malate, mannitol, α/β glucose, oleuropein, and aldehyde derivatives were considered in the graphic summary of the quantitative differences in discriminating metabolites of leaf extracts between treated vs. untreated sample comparisons (Figure 12).

The treated samples of the July 2023 sampling are characterized by high relative concentrations of mannitol and glucose compared to the untreated samples, which show a higher content of oleuropein and aldehyde derivatives.

In the October 2023 sampling, the presence of high relative concentrations of quinic acid and mannitol can be observed for treated samples, while the untreated samples show a higher content of malate, as well as oleuropein and aldehyde derivatives.

Regarding the two 2024 samplings, the results are quite in line with those observed for the 2023 samplings. Indeed, in the July 2024 sampling, the treated samples show a higher content of glucose compared to the untreated samples, which are characterized by increased malate, as well as oleuropein and aldehyde derivatives.

In the October 2024 sampling, the treated samples showed a higher content of mannitol, whereas glucose and malate achieved higher content in untreated samples. Both cumulative yearly and complete dataset comparisons account for the treated vs. untreated fold changes observed in the single samplings.

## 3. Discussion

Among the commercially available chemical treatments suggested for a possible use against OQDS in plants affected by *Xfp* are Dentamet^®^ and NuovOlivo^®^ [27]. It has been reported that six spray treatments a year with Dentamet^®^ (a biocomplex of zinc, copper, and citric acid) decreased serious symptoms related to OQDS and *Xfp* in infected olive trees [10].

On the other hand, according to published results, NuovOlivo^®^, a natural detergent based on vegetable oils and an aqueous infusion of various plants, combined with sodium hydroxide, calcium, and sulphur, could also represent a possible protocol to counteract the symptoms caused by *Xfp* infection. Various preliminary studies demonstrated that infection indices decreased significantly in NuovOlivo^®^-treated plants compared to the controls [6,11,26].

In this work, the effects of NuovOlivo^®^, tested on olive trees naturally affected by Olive Quick Decline Syndrome associated with *Xfp*, were studied through ^1^H-based metabolomics. The study was conducted on seven olive trees naturally infected by *Xfp* and exhibiting OQDS symptoms, located in Montesano Salentino (Lecce province, Salento peninsula, South of Apulia, Italy), treated with the product, as described in the work of Bruno et al. [26], in comparison with the untreated trees. The experiments were conducted to verify, by ^1^H NMR based metabolomics of leaf extracts of the variety Cellina di Nardò, possible metabolic alterations induced by the NuovOlivo^®^ treatment compared to untreated plants. The samplings were carried out in July 2023, and then in October 2023, July 2024, and October 2024.

The study of the variations in the plant’s metabolic profile related to the treatment clearly indicated the need for a careful analysis according to the specific sampling period. Indeed, preliminary unsupervised and supervised analyses of both the complete dataset and the two separate datasets regarding two different years of treated and untreated leaf samples also showed a clear relevance of seasonality for sample group clustering. In particular, the exploratory PCA analysis, conducted on the total samples (Figure 1), highlighted a clear clustering of the July 2024 samples, thus indicating differing variability between July and October samples across the two considered years. In particular, the July 2024 samples are characterized by a higher sugar content, such as mannitol and glucose. Therefore, specific supervised OPLS-DA analyses were performed to discriminate between treated and untreated samples for each of the samplings (July 2023, October 2023, July 2024, October 2024). These single sampling results were then compared to the cumulative (both yearly and complete dataset) findings. Key outcomes could be found in the S-line plots for the treated vs. untreated OPLS-DA models (Figure 2, Figure 3, Figure 4, Figure 6, Figure 7, Figure 9 and Figure 10), compared all together in Figure S3, as well as in the summary of the fold changes for the discriminating metabolites obtained considering the single and cumulative sampling (Figure 12). Moreover, the differences in leaf extracts from treated and untreated samples clearly related to specific pool of endogenous metabolites, as observed for other olive tree xylematic extracts [9,33,34].

The results of the used untargeted ^1^H NMR fingerprinting metabolomics approach with unsupervised and supervised MVA techniques demonstrated that major differences were generally observed for the content of quinic acid, malate, mannitol, glucose, oleuropein, and aldehyde derivatives in treated compared to untreated samples. In particular, a relatively higher content of quinic acid was found in the treated compared to untreated samples essentially due to the significant 2023 October sampling contribution.

On the other hand, a constant mostly significant lower presence of malate and higher of mannitol was found in treated compared to untreated samples for all the sampling and performed comparisons. A relatively higher content of glucose (α and β) was also detected for treated vs. untreated samples in the general dataset comparison as a result of the mostly significant consistent (2023 and July 2024) and adverse (October 2024) sampling contributions.

A significant lower level of oleuropein and aldehyde derivatives was observed in the treated compared to untreated samples comparison of the complete dataset. In this case, specific contributions to the fold changes were consistent in both years but much higher and more significant for the 2023 compared to the 2024 sampling. The decreasing trend (2023 vs. 2024) of higher levels for quinic acid, a precursor of lignin, already reported as a disease biomarker for the olive trees infected by *Xfp* [14], in treated leaf samples, suggests a possible specific impact of the treatment with NuovOlivo^®^, in accordance with other works on the effect of other biocomplex treatments [3,9,30]. The increased production of quinic acid in infected plants could be linked to its involvement in the plant’s secondary metabolism, as this molecule is known to be the precursor of lignin and phenolic and aromatic compounds, indicators of stress or infection in olive trees [17].

On the other hand, as described in other studies, the constantly higher content of the mannitol, observed in treated leaf samples, indicates a protection against stress and an improvement in physiological performance and photosynthetic capacity of olive trees [3,29,30,35]. Moreover, the variation in malate concentrations, with lower levels in treated vs. untreated samples, could suggest increased consumption of this metabolite to support normal citric acid cycle functions and could also be related to the increased plant’s response to stress conditions [9]. As already reported, together with increased mannitol, the elevated glucose’s levels observed in treated compared to untreated samples could indicate a seasonality-linked cascade of defence responses against disease, aimed at increasing carbon uptake [3,9,30,36]. Increased sugar content in treated trees could be further associated with both their osmoprotective function and their biochemical signalling of photosynthetic reactivation.

Finally, the untreated leaf samples extracts are characterized by higher content of oleuropein and aldehyde derivatives, especially observed for the first year of sampling (2023). Polyphenols, such as oleuropein and derivatives, constitute the most abundant and defensive compounds that help olive plants counteract various stresses [35]. The higher relative polyphenol content in untreated trees could suggest a possible natural resilience activated by the plants. Indeed, it is well known that polyphenols are antioxidant and protective compounds, a possible indicator of the activation of a natural physiological compensatory metabolism also in the absence of biostimulant treatment. Interestingly, induced polyphenol production has been clearly demonstrated by ^1^H-NMR monitoring of leaves extracts from *Xfp*-infected olive trees in a specific endo-therapy treatment follow-up [30]. Nevertheless, in the medium to long term, treated plants generally show lower levels of oleuropein and its derivatives compared to untreated plants [9,29]. This occurs besides the increase due to the possible treatment-induced short-term polyphenol production, which parallels the natural plant response to the disease [30]. In accordance with the present study, a relatively higher content of phenolic compounds and aldehydic forms of oleuropein and ligstroside was observed in untreated infected trees of Ogliarola Salentina and Cellina di Nardò when compared to leaf samples treated with Dentamet^®^ [9]. These results were confirmed by subsequent research in which the Dentamet^®^-treated samples of Ogliarola Salentina showed a higher level of mannitol, while untreated samples showed higher relative content of tyrosol/hydroxytyrosol and aldehydic forms of oleuropein [29]. As known, the production of phenolic compounds by plants is a defensive strategy against various pathogens [23], and it has been demonstrated by numerous studies that these molecules can inhibit the growth of various bacterial species [24,37], including *Xfp* in grapevines and almond [38]. In addition, the antibacterial properties of olive leaf extracts with important oleuropein content were examined, observing a significant inhibition of *Xfp* growth [25]. Thus, oleuropein could be considered a powerful natural agent against *Xfp* [11]. On the other hand, as reported by Ugolini et al. in a recent study, the content of oleuropein and other phenolic compounds is influenced by seasonality, cultivars, years, etc. [39]. Indeed, according to the results reported here, the metabolic plant response to both disease and possible control treatments appears also influenced by seasonality and in the case of *Xfp* olive infection, it undoubtedly requires further specific investigations. In the present study, the general examination of the S-line plots reported in Figure 2, Figure 3, Figure 4, Figure 6, Figure 7, Figure 9 and Figure 10 and fold changes summary (Figure 12) clearly suggest that, for both the investigated years: (i) the differences (treated vs. untreated) observed for the July samples appear generally more marked with respect to October; and (ii) the differences observed in the yearly cumulative comparisons appear essentially controlled by those of the October with respect to July sampling. This evidence indicated a season-dependent plant response to both disease and treatment.

The observed variations (e.g., the glucose/mannitol ratio in October 2024) between the 2023 and 2024 harvest year could be explained as metabolic evidence of the plant’s dynamic adaptation/response to both disease and treatment over the considered timespan. The results showed significant differences in the metabolic profiles of leaf extracts from the treated plants compared to the untreated ones, exhibiting season and year dependence. Moreover, the overall differences observed between the two investigated years, as well as the comparison of the cumulative 2023 and 2024 results, suggest a general decrease in the differences for the discriminating metabolites over the time. This could be in accord with a smoothing across the two investigated years, of the differences between treatment induced and natural plant response to the disease, in line with the recently observed resilience phenomena in *Xfp* olive orchards in Salento [40].

## 4. Materials and Methods

### 4.1. Leaf Samples: Origin and Collection

In this research, the NuovOlivo^®^ product was tested on olive (*Olea europaea* L., Oleaceae) trees naturally infected by OQDS associated with *Xfp*. NuovOlivo^®^ (Ministero dello Sviluppo Economico, patent n 102017000109094, Italy [41]) is a plant-derived formulation, known as natural detergent made with plant oils and water infusion from multi botanical species, plus sodium and calcium hydroxide, in addition to sulphur. NuovOlivo^®^ includes *Thymus vulgaris* L., *Petroselinum crispum* (Mill.) Fuss, *Crataegus monogyna* Jacq., *Foeniculum vulgare* Mill., *Rosmarinus officinalis* L., *Salvia officinalis* L., *Origanum vulgare* L., *Matricaria chamomilla* L., *Malva sylvestris* L., *Opuntia ficus*-*indica* (L.) Mill., *Ruscus aculeatus* L., *Salix babylonica* L., *Urtica dioica* L., *Capsicum annuum* L., *Piper*, and other Mediterranean plants [26]. The preparation of NuovOlivo^®^ involves treating various plant tissues at 50 °C to balance the preservation of heat-labile compounds with the enhanced solubility of bioactive components such as anthocyanins and flavonoids [26,41].

The study was conducted on seven olive trees naturally infected by *Xfp*, located in Montesano Salentino (Lecce province, Salento peninsula, South of Apulia, Italy). The geographical coordinates of the field are 39°59′24.3″ N 18°18′06.3″ E. The NuovOlivo^®^ treatment was conducted on the plants (70–75 year-old) of the cultivar Cellina di Nardò, as described in the work of Bruno et al. [26].

The protocol with NuovOlivo^®^ also includes good agricultural practices. The plants are sprayed with 10 L of water containing 2% NuovOlivo^®^ and 1% sodium bicarbonate as an activator added to the solution before application. This protocol has been shown to stimulate the growth of new vegetation and promote the production of flowers, fruits, and oil, possibly helping in preserving the *Xfp* susceptible cultivars that form part of the Apulian olive biodiversity [6].

The experiments were conducted to investigate possible metabolic alterations induced using the NuovOlivo^®^ product on leaf extracts samples of the Cellina di Nardò cultivar obtained from plants affected by *Xfp*, treated and untreated with the product. A total of four consecutive olive leaf samplings, on both treated and untreated (control) trees, were performed. The first olive leaf sampling on plants under treatment with the product, as well as on untreated trees, was carried out in July 2023. Subsequent collections occurred in October 2023, July 2024, and October 2024. For each of the seven olive trees, four treated and three untreated, one mix of leaves (ca 20 mature green leaves/mix) was collected in the four subsequent sampling periods. The collected mix of leaves was carried to the laboratory in a chilled box and kept at −20 °C until extraction for metabolomic analysis. Representative figure of sampling field showing the seven investigated trees (four treated and three untreated) is given in Supplementary Materials (Figures S1 and S2).

### 4.2. Chemicals and Reagents

All chemical reagents for the analysis were of analytical grade. Deuterium oxide (99.9 atom %D) containing 0.05% wt 3-(trimethylsilyl) propionic-2,2,3,3 d4 acid sodium salt (TSP) and potassium phosphate monobasic was purchased from Armar Chemicals (Döttingen, Switzerland). Methanol-d4 (99.9 atom %D), potassium phosphate monobasic were purchased from CARLO ERBA Reagents (Milano, Italy).

### 4.3. Preparation of Leaf Extract Samples for ^1^H NMR Analysis

The samples for ^1^H-NMR were prepared according to the experimental procedure reported in the literature [42]. Three technical replicates were obtained from each of the collected leaf pools for a total of 84 samples (3 technical replicates for each of the 7 studied trees from 4 subsequent sampling periods). The olive leaf samples (each one containing 20 leaves) were plunged into liquid N_2_ and ground in a stainless-steel blender until a fine powder was obtained. Subsequently, the leaf powder was put into a plastic tube and lyophilized for 48 h. For each sample, the lyophilized plant material (100 mg) was added 0.75 mL of CD_3_OD and 0.75 mL of KH_2_PO_4_ buffer in D_2_O (pH 6) containing 0.05% *w*/*v* TSP-d4 in a 2 mL Eppendorf tube. The content of the Eppendorf tubes was mixed for 1 min using a vortex and then sonicated at room temperature for 10 min. The resulting solutions were centrifuged at 17,000 g for 20 min; then, a 5 mm NMR tube was filled with 600 µL of the supernatant [3].

### 4.4. Acquisition and Processing of ^1^H NMR Spectra of Leaf Extracts

All spectra of leaf extracts were acquired at constant temperature (300 K) on a Bruker Avance III 600 MHz Ascend NMR spectrometer (Bruker Italia, Milan, Italy) with a TCI cryoprobe (inverse Triple Resonance Cryoprobe Prodigy), operating at a 600.13 MHz frequency, with a *z*-axis gradient coil and automatic tuning-matching (ATM). Water signal suppression (Bruker pulseprogram zgcppr) was used for the acquisition of the ^1^H NMR spectrum for each sample, with a spectral window of 20.0276 ppm (12,019.230 Hz), 64 scans, and a 90° pulse of 10.24 µs. The standard and Free Induction Decay (FID) processing was performed using TopSpin 3.6.1 (Bruker, Biospin, Italy) after every acquisition with 0.3 Hz line broadening (LB). Therefore, the phase and baseline correction were executed.

The metabolites were identified and assigned through a comparison with the data from the literature [3,9,30]. Chemical characterization of the xylematic extracts (Table S1) revealed a profile consistent with the previously described composition of olive leaves [9,33,34], confirming that no residual metabolites from the plants used in the formulation remained on the treated leaves.

After data processing, ^1^H-NMR spectra were segmented in rectangular buckets (0.04 ppm width) and integrated using the Bruker Amix 3.9.14 (Analysis of Mixture, Bruker BioSpin GmbH, Rheinstetten, Germany) software. Specifically, each NMR spectrum was segmented into regions or histograms with a fixed base width of 0.04 ppm, known as “normal rectangular bucketing”. The residual non-deuterated water (5–4.65 ppm) and methanol (3.33–3.25 ppm) spectral regions were excluded from the buckets. The reduced spectra and the remaining buckets in the range of 10.00–0.50 ppm were then normalized to total intensity and mean-centred to reduce the variability and noise in the data. The Pareto scaling method was further applied [43]. Therefore, the resulting data table was used in the multivariate data analysis (MVA).

### 4.5. Statistical Analysis Applied to Acquired Data

After the acquisition and processing of the ^1^H NMR spectra of the leaf extracts, the multivariate statistical analysis (MVA) was performed using the Simca-P, version 14 (Sartorius Stedim Biotech, Umeå, Sweden), software.

Unsupervised (Principal Component Analysis, PCA) and supervised (Orthogonal Partial Least Square Discriminant Analysis, OPLS-DA) methods were applied to explore the intrinsic data variation. PCA constitutes the first chemometric step in data analysis aimed at extrapolating the maximum possible information and providing a general description of the natural sample distribution [44,45].

Subsequently, supervised analysis (OPLS-DA) was used to maximize the separation between the classes of samples. OPLS-DA is a powerful method for the interpretation and classification of sample dataset, also providing information on the variables (NMR data) responsible for the classes’ separation [46,47,48]. The models were validated through the internal cross-validation default method (7-fold) and permutation test. The R^2^ and Q^2^ parameters described the quality of the models. The R^2^ is a cross-validation parameter and indicates the goodness of fit. The Q^2^ indicates the portion of data variance predictable by the model [48,49,50].

The tools available on the SIMCA-P software (loading scatter plot and S-line plot) were used to identify the metabolites responsible for the class sample discrimination. The loading scatter plot and S-line plot, applied to PCA and OPLS-DA models, respectively, were coloured according to the absolute value of the correlation, p(corr). The colour bar of the plot describes the correlation of the segregated metabolites between groups [49,51,52].

The log2–FC ratio of the normalized median intensity of corresponding selected bucket-reduced unbiased NMR signals was calculated to gain the relative quantification of discriminating metabolites [3,52]. Moreover, only metabolites with a VIP (variable importance in projection), estimated from all extracted components, and a correlation coefficient, p(corr), respectively, with absolute values higher than 1.0 and 0.5 were considered as possible significant discriminants and considered for the relative quantification [53].

## 5. Conclusions

In this study, we characterize, for the first time by ^1^H NMR-based metabolomics, the potential effects on the metabolic profiles of *Xfp* naturally infected Cellina di Nardò olive trees in Salento (Italy), treated with the natural plant-derived formulation, NuovOlivo^®^. The results showed significant differences in the metabolic profiles of leaf extracts from treated plants compared to the untreated ones, exhibiting season and year dependence. The observed differences could be related to specific plant metabolic responses, involving key metabolites associated with the activation of infection resistance mechanisms. The reported general decrease in the differences for the discriminating metabolites between the two investigated years suggest a smoothing over the time between treatment induced and natural plant response to the disease, consistently to the recently reported *Xfp* olive orchard resilience phenomena. These results support previous studies showing improved vegetative growth and a reduction in symptoms in NuovOlivo^®^-treated *Xfp* infected olive trees compared to the untreated controls. Indeed, the protocol NuovOlivo^®^ could trigger a specific metabolic reprogramming in infected olive trees, providing novel insights into the management of *Xfp* and OQDS and suggesting a possible role of this treatment, integrated with good agricultural practices, in olive grove recovery.

The observed differences could be related to specific plant metabolic responses involving the activation of infection resistance mechanisms.

## Data Availability

The data are contained within the article and in the Supplementary Materials. Further inquiries can be directed to the corresponding author.

## Supplementary Materials

The following supporting information can be downloaded at: https://www.mdpi.com/article/10.3390/plants15071109/s1, Figure S1: Visual records of olive trees during the 2023 seasonal samplings (July and October).; Figure S2: Visual records of olive trees during the 2024 seasonal samplings (July and October). Figure S3: Overview of the whole S-line plots for the treated vs. untreated OPLS-DA models (Figure 2, Figure 3, Figure 4, Figure 6, Figure 7, Figure 9 and Figure 10). Table S1. ^1^H-NMR assignments of main olive leaf metabolites. Detected metabolites are in bold.

## Abbreviations

The following abbreviations are used in this manuscript:

Co.Di.R.O	Complesso del Disseccamento Rapido dell’Olivo
NMR	Nuclear Magnetic Resonance
MVA	multivariate statistical analysis
OPLS-DA	Orthogonal Partial Least Square Discriminant Analysis
OQDS	Olive Quick Decline Syndrome
PCA	Principal Component Analysis
*Xfp*	*Xylella fastidiosa* subsp. *pauca*
