# Supplementary material for: 1H NMR Approach for Evaluating the Effects of a Natural Detergent on Olive Trees Infected by Xylella fastidiosa subsp. pauca
Source: Plants (Basel). 2026 Apr 3;15(7):1109. doi: 10.3390/plants15071109 (PMC13074645; doi:10.3390/plants15071109)
Supplement: Supplementary file 1 [file plants-15-01109-s001.zip › plants-4177048-supplementary.pdf]

plants

MDPI

Supplementary Materials

^{1}H NMR Approach for Evaluating the Effects of a Natural Detergent on Olive Trees Infected by *Xylella fastidiosa* subsp. *pauca*

Miriana Carla Fazzi [1], Chiara Roberta Girelli [1*], Francesco Paolo Fanizzi [1,]

[1] Department of Biological and Environmental Sciences and Technology, University of Salento, 73100 Lecce, Italy; mirianacarla.fazzi@unisalento.it (M.C.F.); fp.fanizzi@unisalento.it (F.P.F.).
* Correspondence: chiara.girelli@unisalento.it (C.R.G.).

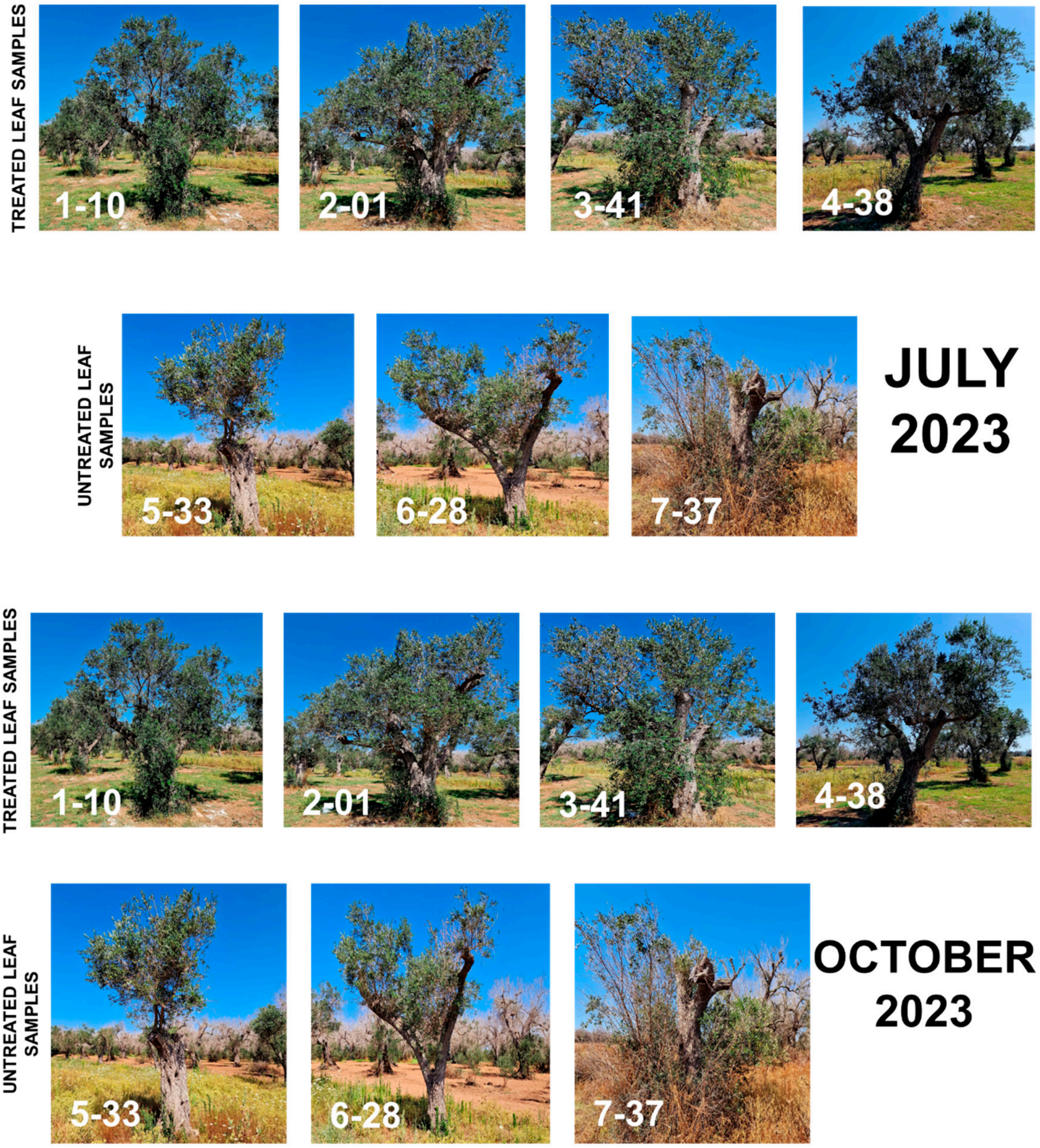

Figure S1. Visual records of olive trees during the 2023 seasonal samplings (July and October). Seven olive trees of the cultivar Cellina di Nardò, naturally infected by *Xfp*, on which this study was conducted. The four samples treated with NuovOlivo® correspond to the trees with code 1-10, 2-01, 3-41, 4-38, while the three samples untreated correspond to the trees with code 5-33, 6-28, 7-37. All plants are located in Montesano Salentino (Lecce province, Salento peninsula, South of Apulia, Italy).

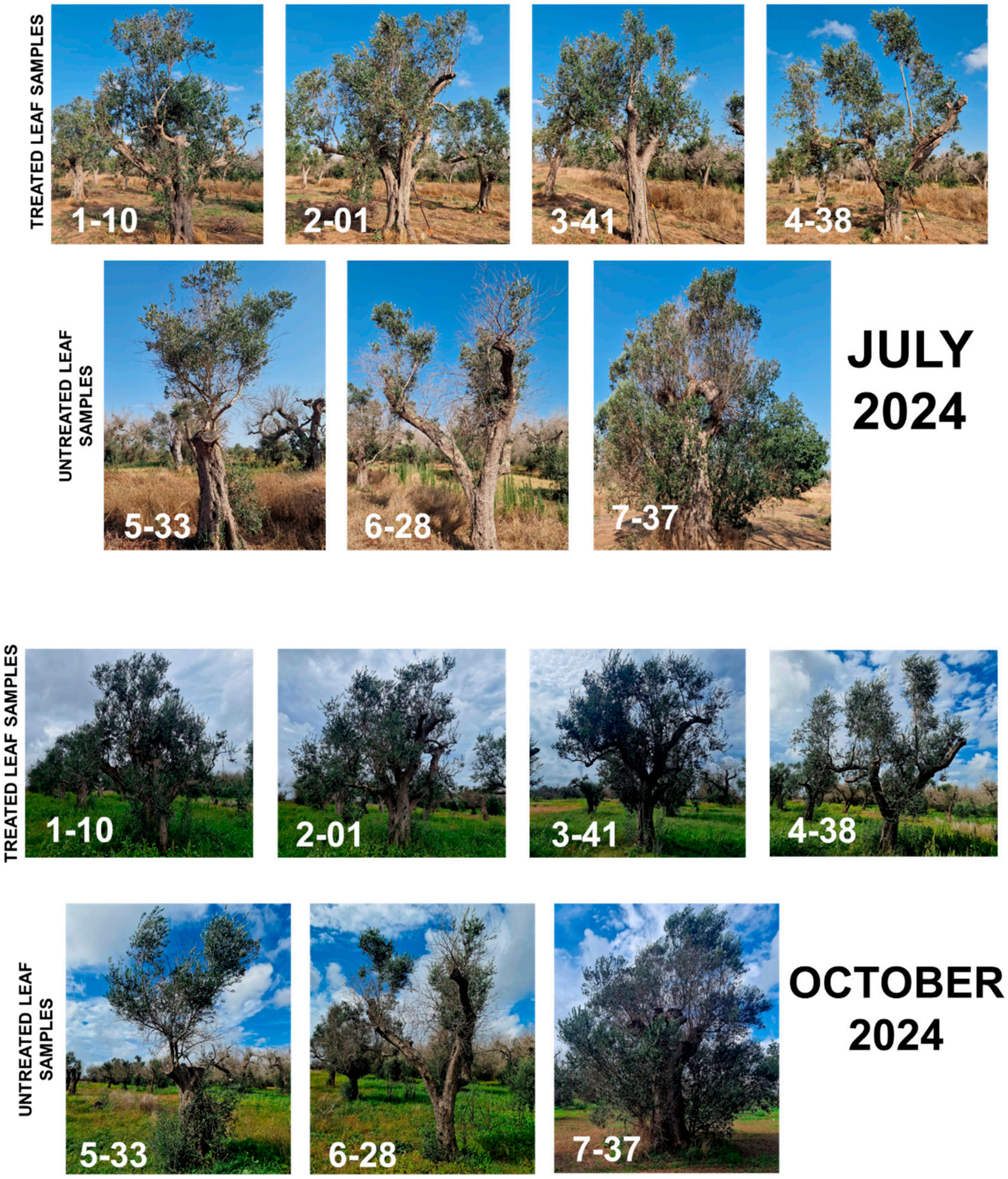

Figure S2. Visual records of olive trees during the 2024 seasonal samplings (July and October). Seven olive trees of the cultivar Cellina di Nardò, naturally infected by *Xfp*, on which this study was conducted. The four samples treated with NuovOlivo® correspond to the trees with code 1-10, 2-01, 3-41, 4-38, while the three samples untreated correspond to the trees with code 5-33, 6-28, 7-37. All plants are located in Montesano Salentino (Lecce province, Salento peninsula, South of Apulia, Italy).

https://doi.org/10.3390/plants15071109

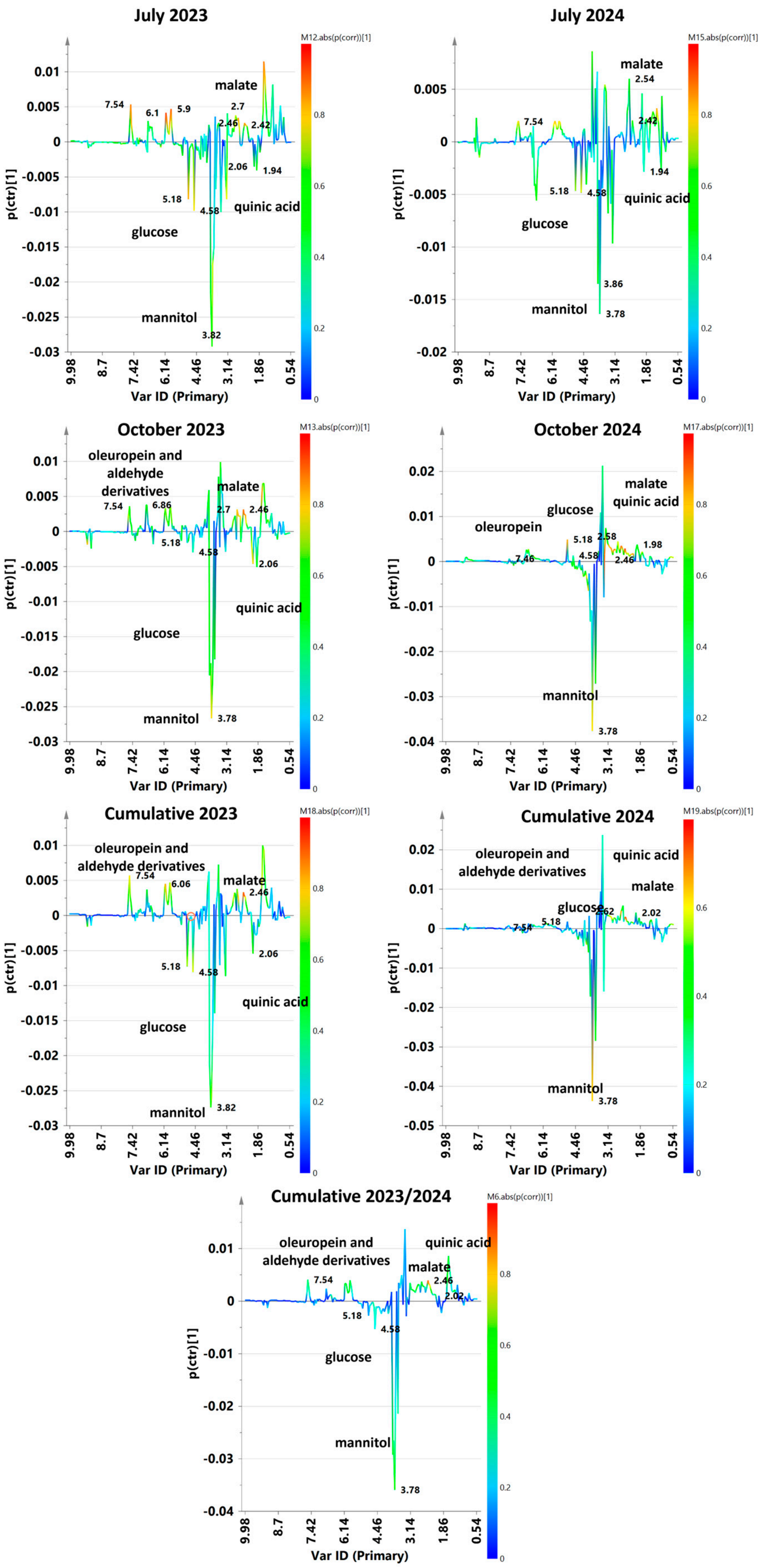

Figure S3. Overview of the whole S-line plots for the treated vs untreated OPLS-DA models (Figure 2, 3, 4, 6, 7, 9, 10).

Table S1. ¹H-NMR assignments of main olive leaves metabolites. Detected metabolites are in bold.

**Metabolite**	**Chemical Shift (ppm)** [1–4]
Maslinic acid and oleanolic acid	0.77; 0.96
**Alanine**	**1.51**
**Quinic acid**	**[2.05-2.00]; [1.90-1.86]**
**Acetate**	**1.9**
**Malate**	**2.70; 2.46**
Succinate	2.40
**Choline**	**3.20**
**Mannitol**	**[3.66-3.84]**
**α-Glucose**	**5.18, 3.50**
**β-Glucose**	**4.57, 3.20**
**Sucrose**	**5.41**
**Hydroxytyrosol**	**6.90 - 6.70**
**Tyrosol**	**6.84 - 6.70**
**Oleuropein**	**7.55; 6.06, 5.91; 5.85; 1.60**
Verbascoside	6.31; 6.63; 6.61;
**Aldehydic and dialdehydic forms of oleuropein and ligstroside**	**9.27; 9.13**

References

1. Girelli; Angilè; Del Coco; Migoni; Zampella; Marcelletti; Cristella; Marangi; Scortichini; Fanizzi 1H-NMR Metabolite Fingerprinting Analysis Reveals a Disease Biomarker and a Field Treatment Response in Xylella Fastidiosa Subsp. Pauca-Infected Olive Trees. *Plants* **2019**, *8*, 115, doi:10.3390/plants8050115.
2. Jlilat, A. A Non-Targeted Metabolomics Study on Xylella Fastidiosa Infected Olive Plants Grown under Controlled Conditions. *Scientific Reports* **2021**, 11.
3. Tatini, D.; Bisozzi, F.; Costantini, S.; Fattori, G.; Boldrini, A.; Baglioni, M.; Bonechi, C.; Donati, A.; Tozzi, C.; Riccaboni, A.; et al. Geographical Origin Authentication of Leaves and Drupes from Olea Europaea via 1H NMR and Excitation–Emission Fluorescence Spectroscopy: A Data Fusion Approach. *Molecules* **2025**, *30*, 3208, doi:10.3390/molecules30153208.
4. Girelli, C.R.; Del Coco, L.; Scortichini, M.; Petriccione, M.; Zampella, L.; Mastrobuoni, F.; Cesari, G.; Bertaccini, A.; D'Amico, G.; Contaldo, N.; et al. Xylella Fastidiosa and Olive Quick Decline Syndrome (CoDiRO) in Salento (Southern

Italy): A Chemometric 1H NMR-Based Preliminary Study on Ogliarola Salentina and Cellina Di Nardò Cultivars. *Chem. Biol. Technol. Agric.* **2017**, *4*, 25, doi:10.1186/s40538-017-0107-7.
